# Dissociation of Regional Activity in Default Mode Network in Medication-Naive, First-Episode Somatization Disorder

**DOI:** 10.1371/journal.pone.0099273

**Published:** 2014-07-01

**Authors:** Qinji Su, Dapeng Yao, Muliang Jiang, Feng Liu, Jiajing Jiang, Chunxing Xu, Yi Dai, Miaoyu Yu, Liling Long, Hongzheng Li, Jianrong Liu, Zhikun Zhang, Jian Zhang, Changqing Xiao, Wenbin Guo

**Affiliations:** 1 Mental Health Center, the First Affiliated Hospital, Guangxi Medical University, Nanning, Guangxi, China; 2 Department of Radiology, the First Affiliated Hospital, Guangxi Medical University, Nanning, Guangxi, China; 3 Key Laboratory for NeuroInformation of Ministry of Education, School of Life Science and Technology, University of Electronic Science and Technology of China, Chengdu, Sichuan, China; 4 Mental Health Institute, the 303rd Hospital of Chinese people's Liberation Army, Nanning, Guangxi, China; University of Electronic Science and Technology of China, China

## Abstract

**Background:**

Patients with somatization disorder (SD) have altered neural activity in the brain regions of the default mode network (DMN). However, the regional alteration of the DMN in SD remains unknown. The present study was designed to investigate the regional alterations of the DMN in patients with SD at rest.

**Methods:**

Twenty-five first-episode, medication-naive patients with SD and 28 age-, sex-, education- matched healthy controls underwent a resting-state functional magnetic resonance imaging (fMRI) scan. The fractional amplitude of low-frequency fluctuations (fALFF) was applied to analyze the data.

**Results:**

Patients with SD showed a dissociation pattern of resting-state fALFF in the DMN, with increased fALFF in the bilateral superior medial prefrontal cortex (MPFC, BA8, 9) and decreased fALFF in the left precuneus (PCu, BA7). Furthermore, significantly positive correlation was observed between the z values of the voxels within the bilateral superior MPFC and somatization subscale scores of the Symptom Check List (SCL-90) in patients with SD.

**Conclusions:**

Our findings indicate that there is a dissociation pattern of the anterior and posterior DMN in first-episode, treatment-naive patients with SD. The results provide new insight for the importance of the DMN in the pathophysiology of SD.

## Introduction

Somatization disorder (SD) is an illness with lots of physical complaints in a variety of systems that occurs at least 2 years resulting in significant impairment or treatment seeking. The somatic symptoms typically refer to gastrointestinal, cardio-respiratory, uro-genital, other internal systems, or musculoskeletal problems. The disorder usually begins before age 30 and occurs more often in women than in men (about 1∶5, men to women). The prevalence of SD in the general population may be around 4–7% [1]–[4]. Patients with SD suffer significantly psychosocial disability leading to a high rate of health care utilization, a decline of the life quality, absenteeism from work and reduction in productivity [5], [6]. Health care burden of SD is comparable to that of some severe mental illnesses like schizophrenia and depression [7]. Although the pathophysiology of SD remains unclear, the progress in neuroimaging technology makes it possible to study the neurobiology of SD [8].

Somatization accompanied by anxiety has been associated with decreased metabolism in some brain regions, including ventrolateral prefrontal cortex, ventral anterior cingulate gyrus (ACC) and anterior insular gyrus [9]. In a structural magnetic resonance imaging (MRI) study, patients with SD had significantly smaller mean volumes in amygdala compared to healthy controls [10]. Moreover, Fayed et al. found that enhanced activity in posterior cingulate cortex (PCC) in SD patients relative to controls [11]. Lemche et al. reported that when SD patients were in different emotional states (happy or sad), abnormal activity existed in the anterior ventral precuneus (PCu, BA7), posterior cingulate gyrus and anteromedial thalamus [12]. These findings provide evidence of neuroanatomical and neurophysiologic alterations in patients with SD. Recently, the default mode network (DMN) attracts more attention in the study of psychiatric disorders. The DMN, consisting of medial prefrontal cortex (MPFC), ACC, PCC/PCu and dorsomedial thalamus, exhibits high levels of activity at rest and becomes suspended or deactivated when specific goal-directed behavior is needed [13]–[17]. Furthermore, a recent systematic and critical review suggests that the DMN may act as an important role in the pathophysiology of SD [18]. We endorsed these studies for that they provide useful clues with altered activity of the DMN in SD. However, there is still lack of evidence about the changes of regional activity in SD.

The fractional amplitude of low-frequency fluctuations (fALFF), which is measured with resting-state functional magnetic resonance imaging (fMRI), is one of such methods to detect regional signal changes of spontaneous activity. The fALFF approach, an improved index of amplitude of low-frequency fluctuations (ALFF), is concerned with the percentile of the power spectrum in low-frequency (e.g.0.01–0.08 Hz) oscillations (LFOs) versus that of the entire detectable spectrum (e.g.0–0.25 Hz). In contrast to ALFF, fALFF can provide a higher specific measure of LFOs and effectively reflects intrinsic neural activity and physiological states within specific regions. Therefore, non-specific signal components could be effectively suppressed by this technology, and the sensitivity and specificity in examining regional spontaneous brain activity could be significantly improved [19], [20]. So far, this method has been successfully used to investigate the brain function in healthy subjects [21] and clinical populations [22]–[28].

The aim of the current study was to examine regional neural activity of the brain in SD patients by using the fALFF approach. According to the aforementioned studies [9]–[12], [18], which provide useful clues with altered activity of the DMN in SD. Therefore, we hypothesized that (1) the differences of fALFF exist in the DMN between the patients and the controls; (2) the differences of fALFF were expected to be related to some clinical variables, such as the somatization subscale of the Symptom Check List 90 (SCL-90) [29], the Hamilton depression scale (HAMD) [30] and the Hamilton anxiety scale (HAMA) [31] in the patient group.

## Materials and Methods

### Subjects

Twenty-six right-handed patients with SD, originally recruited from the Mental Health Center, the First Affiliated Hospital of Guangxi Medical University, China, participated in the whole study. The patients were recruited consecutively and diagnosed according to the Structured Clinical Interview of the DSM-IV (SCID) [32]. All patients were at their first episode and medication-naive. Exclusion criteria included age younger than 18 years or older than 60 years, history of loss of consciousness, mental retardation, cardiovascular disease, bipolar disorder, neurological illness, and alcohol or drug abuse. However, comorbidity with major depressive disorder (MDD) is allowable.

Thirty right-handed healthy controls were recruited from the community. None of them had a history of serious medical or neuropsychiatric illness, craniocerebral operations, a family history of major psychiatric or neurological illness in their first-degree relatives, and all were matched with the patients in terms of age, sex ratio and years of education.

All subjects were assessed with the HAMD, HAMA, somatization subscale of the SCL-90 at the scan day. The somatization subscale of SCL-90, consisting of 12 physical symptoms items (e.g. headaches, dizziness, pains in lower back, nausea, etc.), was used to assess the somatic symptom severity. The 17-item HAMD and 14-item HAMA, respectively, were used to measure the depressive symptom and the anxious symptom. The three scales were used widely in psychiatric research. The reliability and validity of the three scales are all above 0.8.

The study was approved by the Ethics Committee of the First Affiliated Hospital, Guangxi Medical University. All participants were given information about the procedures and signed an informed consent.

### Imaging acquisition

Imaging was acquired by a 3.0 T Siemens scanner (Siemens, Erlangen, Germany) located in the First Affiliated Hospital, Guangxi Medical University, China. To minimize head movement and reduce scanner noise, foam padding and earplugs were used. During the scanning, subjects were instructed to lie still with their eyes closed and remain awake. Functional images were obtained with an echo-planar imaging (EPI) sequence. The following parameters were used for functional imaging as follows: repetition time/echo time (TR/TE) = 2000/30 ms, slices = 30, thickness = 4 mm, gap = 0.4 mm, field of view (FOV) = 24 cm, flip angle = 90°, data matrix = 64×64. For each subject, the fMRI scanning lasted for 500 s and 250 volumes were obtained.

### Data preprocessing

Image preprocessing was conducted using statistical parametric mapping software (SPM8, http://www.fil.ion.ucl.ac.uk/spm). The first 10 volumes of each subject were discarded due to the signal reaching equilibrium and the participants adapting to the scanning noise. The remaining 240 volumes were corrected for acquisition delay between slices and for head motion. The subjects should have no more than 2 mm maximum displacement in x, y, or z and 2° of angular motion during the whole fMRI scan. The resulting images were spatially normalized to the Montreal Neurological Institute (MNI) EPI template in SPM8, and each voxel was resampled to 3×3×3 mm^3^. After this, the processed images were spatially smoothed with an 8 mm full width at half maximum (FWHM) Gaussian kernel.

### fALFF data analysis

After preprocessing in SPM, linear trend was removed and fALFF analysis was performed using the REST software [33]. Briefly, the time course of each voxel was first converted to the frequency domain without band-pass filtering by using a Fast Fourier Transform (FFT) and the power spectrum was obtained. Since the power of a given frequency was proportional to the square of the amplitude of its frequency component, the square root was calculated at each frequency of the power spectrum and the averaged square root was obtained across 0.01–0.08 Hz at each voxel. The sum of amplitude across 0.01–0.08 Hz was divided by that across the entire frequency range. For a standardization purpose, the fALFF of each voxel was divided by the global mean fALFF value within a brain mask.

### Statistical analysis

**Figure 1:**
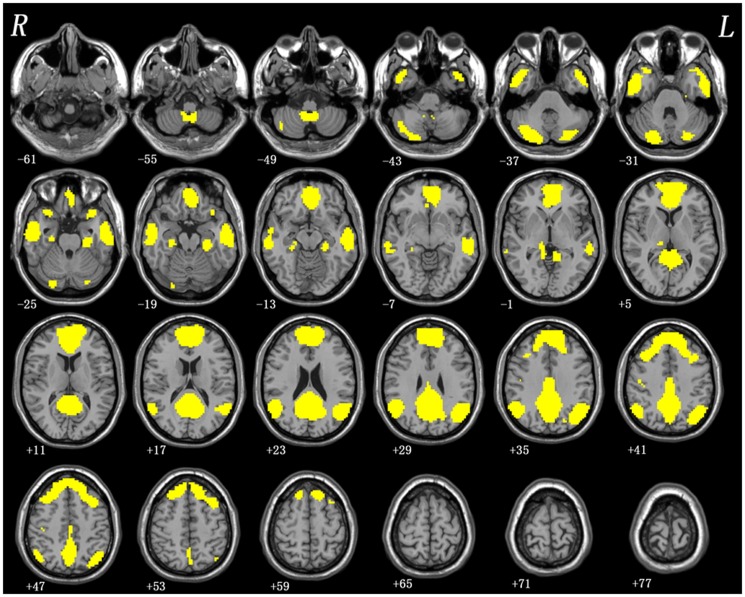
The DMN mask produced in our previous studies. DMN = default mode network.

After assessing normal distributions, the fALFF analyses were performed with the independent two-sample t-tests via voxel-wise cross-subject statistics in the regions within the DMN mask, which was produced in our previous studies [34], [35] (see Figure 1). The resulting statistical map was corrected for multiple comparisons to a significant level of *p*<0.05 after AlphaSim correction (combined with the individual voxel *p*<0.01 and cluster size >52 voxels). This correction was confined within the DMN mask and determined by Monte Carlo simulations using the AFNI AlphaSim program (http://afni.nih.gov/afni/docpdf/AlphaSim.pdf). After testing the normality, voxel-based correlation analyses were implemented (*p*<0.05) to identify the relationship of the fALFF values in regions with significant group differences and the severity of anxiety, depression or somatic symptoms. The results were constrained within the DMN mask. The statistical results were corrected using AlphaSim program in the Resting-State fMRI Data Analysis Toolkit (REST) software [33] at a significant level of *p*<0.05 (combined height threshold *p*<0.01 and a minimum cluster size >52). The demographic and clinical data were compared by using two-sample t-test and Chi-square test (*p*<0.05).

## Results

### Demographics and clinical characteristics of the subjects

The data of three subjects (one patient and two healthy controls) were discarded from further analysis because of excessive head movement. The demographic and clinical data are presented in Table 1. The two groups were matched in age, sex ratio, and years of education. Compared with healthy control, patients with SD showed higher levels of depression, anxiety and somatic symptoms (*p*<0.001).

**Table 1:** Characteristics of the patients and healthy controls.

Variables (mean ±standard deviation)	Patients	Controls	*p* value
Gender (male/female)	4/21	6/22	0.732^a^
Age, years	41.00±10.76	38.71±9.59	0.417^b^
Education, years	7.72±4.39	7.82±2.59	0.920^b^
Illness duration, months	59.12±62.22		
HAMD	18.84±7.31	2.60±1.83	<0.001^b^
HAMA	22.96±10.95	0.53±0.99	<0.001^b^
somatization subscale of SCL-90	28.48±10.37	14.32±3.44	<0.001^b^

HAMD: Hamilton depression scale; HAMA: Hamilton anxiety scale; SCL-90: Symptom Check List-90.

a The *p* value for gender distribution in the two groups was obtained by chi-square test.

b The *p* values were obtained by two sample *t*-tests.

### Differences in fALFF values between patients with SD and controls

**Figure 2:**
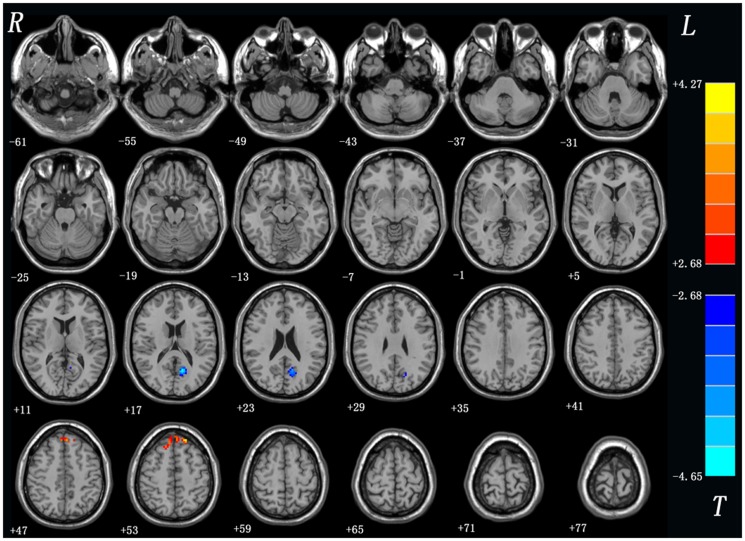
fALFF differences between the patients with SD and the controls. Red and blue denote higher and lower fALFF respectively and the color bars represent the *t* value of the group analysis. fALFF = fractional amplitudes of low-frequency fluctuations; SD = somatization disorder.

As shown in Table 2 and Figure 2, the SD group exhibited significantly increased fALFF in the bilateral superior MPFC (BA8, 9) and decreased fALFF in left PCu (BA7) relative to the controls. No other difference was observed in the patients.

**Table 2:** Regions showing fALFF differences between groups.

Brain regions	Voxels	MNI coordinates (mm)			*t* value
		x	y	z	
SD>controls					
bilateral superior medial frontal gyrus	67	−18	42	54	4.27
SD<controls					
left precuneus	75	−12	−63	18	−4.64

x, y, z, coordinates of primary peak locations in the MNI space; *t* statistical value of peak voxel showing fALFF differences between the patients with SD and the controls. SD: somatization disorder.

### Correlations between the z values of the brain regions and HAMD, HAMA, somatization subscale of SCL-90 in patients

**Figure 3:**
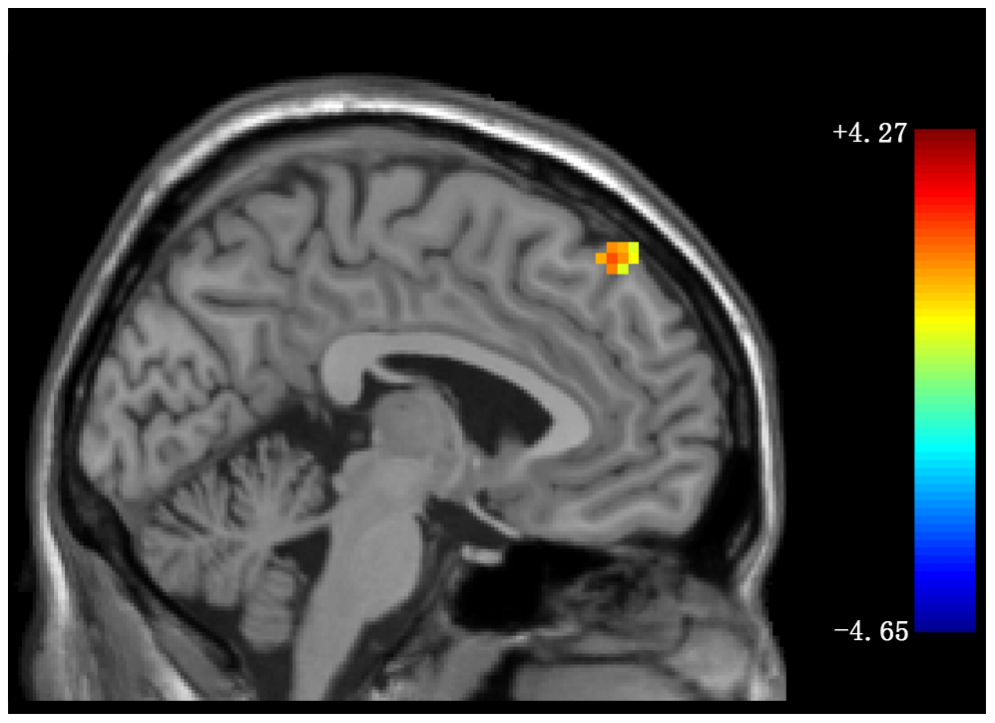
Voxel-wise correlation between the MPFC and the somatization subscale scores of SCL-90 in the patients. Sagittal view of the voxel-wise correlation analysis between z values of the voxels within the the bilateral superior MPFC (Montreal Neurological Institute coordinates: x = −4, y = 42, z = 52) and the somatization subscale scores of SCL-90 in the patient group at 0.05 significance level (combined height threshold *p*<0.01 and a minimum cluster size = 52). MPFC = medial prefrontal cortex; somatization subscale of SCL-90 = somatization subscale of the Symptom Check List.

Significantly positive correlation was observed between the z values of the voxels within the bilateral superior MPFC and the somatization subscale scores of SCL-90 at 0.05 significance level (combined height threshold *p*<0 .01 and a minimum cluster size = 52) (see Figure 3) in the patient group. No significant correlation was found between the z values of other brain regions and the scores of HAMD and HAMA in patients.

## Discussion

Using the fALFF method, the present study demonstrated that fALFF abnormalities of the DMN in medication-naive, first-episode patients with SD. Compared to healthy controls, the patients showed higher fALFF values in the bilateral superior MPFC, and lower fALFF values in the left PCu. Our findings provided evidence of aberrant regional activities in the DMN in patients with SD.

MPFC and PCu are recognized as the core regions of the DMN. Anatomically, MPFC is consisted of cytoarchitectonically discrete areas that receive a wide range of sensory information from the body and the external environment via the orbital prefrontal cortex [36]–[38]. Moreover, MPFC has wide connections among affective-limbic areas (such as the amygdala, hippocampus, and hypothalamus), executive control and emotional processing areas (such as orbital frontal cortex, ACC) [39]. Such anatomical relationships support a role for these medial areas in the integration of the visceromotor aspects of emotional processing with information gathered from the internal and external environments. It is assumed that MPFC plays an important role in emotional processing, such as attention to emotion, identification, or regulation of emotion [40], [41]. Some researchers proposed that MPFC plays a role in the integration of emotional and cognitive processes by incorporating emotional biasing signals or markers into decision-making process [42]–[44]. The different spontaneous neural activity in MPFC may result in the dysfunction of this region, and lead to a loss of top-down regulation, which is regarded as the basis of the pathophysiology of emotional, cognitive and behavioral changes in SD. In somatoform pain disorder patients, MPFC has been found to be dysfunctional. A pain related to hypoactivity of MPFC was found in the somatoform pain disorder patients when under the same pain intensity and pain unpleasantness [45]. It suggests that dysfunction of MPFC may be correlated to somatic pain. Additionally, the correlation between MPFC and pain catastrophizing is supported by neuroimaging data in subjects with chronic pain (fibromyalgia) [46]. Consistent with its key role in both emotion and cognition, we found that increased fALFF existed in MPFC in SD patients, and the z values of the voxels within the MPFC was positively correlated to the somatization subscale scores of SCL-90. The significantly positive correlation suggests that fALFF in MPFC may be applied as an indicator for the somatic symptom severity. Our study thus extends the prior findings by providing new evidence that different regional activity of MPFC may be involved in the pathophysiology of SD.

PCu, which locates in the posteromedial cortex of the parietal lobe, has traditionally received little attention mainly because of its hidden location and rarely lesioned in strokes or accidents. However, its strategic location and wide-spread connections suggest that PCu is a major association area that may subserve a variety of fundamental cognitive and behavioral functions, including visuo-spatial imagery, episodic memory retrieval, self-processing operations and consciousness [47], [48]. It is noteworthy that PCu plays a key role in implementation of a wide range of higher-order cognitive functions. An fMRI study by Knauff et al. investigated the PCu was linked to the cognitive processes of mental imagery in deductive reasoning [49]. PCu has wide-spread associations with cortical and subcortical structures, indicating the complexity of its behavioral specializations. Gusnard et al. suggested that PCu and interconnected medial prefrontal cortices and PCC were engaged in continuous information gathering and representation of the self and external world [50]. Furthermore, PCu and prefrontal cortex are strongly interconnected, and these projections tend to concentrate at the level of BA 8, 9 [51]–[53]. Our findings showed that different neural activity was found in PCu and MPFC in SD patients. The interconnection between PCu and MPFC in SD patients is unclear. However, reduced PCu activation was observed to lead to a loss of MPFC deactivation in Parkinson's disease [54]. Hence, decreased fALFF in PCu in the current study suggests that PCu acts as a role in the pathophysiology of SD.

Previously, an anterior and posterior subsystem of the DMN was identified [55]–[57]. The anterior DMN have been detected hyperactivity in healthy individuals when under pain stimuli [58]. In an fMRI study, the chronic pain disorders showed higher power spectra in the anterior DMN [59]. Our study showed a dissociation pattern between the anterior and posterior parts of DMN, with hyperactivity in anterior regions of the DMN (bilateral superior MPFC) and hypoactivity in the posterior regions of the DMN (left PCu). The role of the DMN is known to participant in self-referential activity, episodic memory retrieval, motivation, cognition, and emotion modulation [13], [60]–[62]. The dissociation pattern of the DMN may result in somatic symptom complex and emotional, cognitive, behavioral changes.

In addition to a relatively small sample size, other limitations should be considered in explaining the results. First, high level of depression and anxiety was found in patients. It is well known that comorbidities existed among depression, anxiety and somatization with a high ratio [63]–[66]. Depression and anxiety may be inherent characteristics of SD, which can not be removed in the analysis. For the same reason, depression and anxiety might have biased the present findings. Second, due to the fact that SD is common in the females, a high proportion of females were enrolled in the present study, which might limit the generalizability of our results. Third, the present study mainly focused on the DMN. It is helpful for understanding the pathophysiological contribution of the DMN. For the same reason, some meaningful findings from other brain regions might be neglected. Fourth, the fALFF method applied in the present study has its own limitations. Although fALFF is regarded as representing regional signal dynamics, the exact physiological nature of fALFF is not entirely clear. Finally, the effects of instrument noise during resting fMRI scans could not be avoided. The noise would pass the LFOs (0.01–0.08 Hz) and influence our findings [67]. In future study, a more rigorous method should be explored to remove such noises.

## Conclusions

Despite the limitations, our findings indicate that there is a dissociation pattern of the anterior and posterior DMN in first-episode, treatment-naive patients with SD. The results provide new insight for the importance of the DMN in the pathophysiology of SD.
